# Adaptation, coping strategies and resilience of agricultural drought in South Africa: implication for the sustainability of livestock sector

**DOI:** 10.1016/j.heliyon.2021.e08280

**Published:** 2021-10-28

**Authors:** Yonas T. Bahta, Vuyiseka A. Myeki

**Affiliations:** Department of Agricultural Economics, University of the Free State, Bloemfontein, South Africa

**Keywords:** Sustainability, Resilience, Adaptation, Agricultural drought, Coping strategy, Smallholder farmer, Livestock

## Abstract

Agricultural drought has put sub-Saharan African under significant pressure, and without adaptation, will negatively influence a future generation. Hence, it is crucial to assess the adaptation and coping strategies, the resilience of agricultural drought, its implication on the sustainability of the livestock sector, and developing future interventions. Data of 217 smallholder livestock farmers were used in a principal component analysis to estimate the agricultural drought resilience index as an outcome variable against social wellbeing, economic outcome, environmental variable and adaptive capacity variables. The results found that 21% of the livestock farming households sold their livestock as an adaptation and coping strategy. In contrast, 20% of the farming households used alternative land use as an adaptation, and coping strategy, 20% stored food, 17% asked for animal feed, 6% sought employment, 6% migrated, 5% kept drought-tolerant breeds, 3% received relief grants, 2% used their savings and investments, and 1% leased their farms. When natural, economic and social sustainability was viewed as a resilience process, the three pillars positively and significantly impacted households' agricultural drought resilience. This implied that the more smallholder farmers participated in social networks and cooperatives, the higher the resilience to agricultural drought. Further, the more resources, income, access to land, access to water, access to credit, and additional types of farming, the higher the households’ resilience to agricultural drought and adaptive capacity. Thus, the three pillars of sustainability are crucial for enhancing the resilience and adaptability of smallholder livestock farmers. The study recommends that government aid reduce vulnerability to agricultural drought via access to agricultural credit and encourage farmers to be part of social networks and cooperatives. Additionally, the government could improve access to land and water rights to boost the resilience of smallholder farmers to agricultural drought. This could be achieved through collaboration and coordination among all role players.

## 1 Introduction

In comparison to floods, hurricanes, tornadoes, and earthquakes, agricultural drought is the costliest natural disaster on the planet. Drought affects the livelihood of the developing world's farmers and economies, where an estimated 166 billion USD loss was recorded from three-quarters of the global cropped area of 454 million hectares. Globally averaged, one drought event decreases agricultural gross domestic production by 0.8%, with varying magnitudes by country (Kim et al., 2019). The impact of agricultural drought puts additional strain on already scarce resources and their long-term sustainability. Africa is the most vulnerable to adapt to the effects of agricultural drought due to limited resources. In developing countries, agricultural drought causes 80% of economic losses and affects the sustainability of agriculture by reducing social wellbeing as well as decreasing economic and environmental resources (Desanker et al., 2001; IPCC, 2001; Wilhite, 2007). Agricultural drought-induced changes to social, economic, and environmental resources affect the livelihoods of smallholder farmers unless adequate measures are taken through adaptation and coping strategies (Osbhar, 2007). The adaptation and coping strategies, agricultural drought resilience, and the sustainability of the agricultural sector, including livestock sectors, depend on economic, social, institutional, environmental, and community factors (Fahad et al., 2017).

Smallholder livestock farmers mainly depend on natural resources such as rainfall, which are directly affected due to climate variability. Besides the shortage of rain, other factors also contribute to the vulnerability of smallholder farmers, such as frequent disasters, poverty, environmental degradation, limited formal safety nets, limited adaptive capacity, limited resources, and weak infrastructure (Shiferaw et al., 2014; Myeki and Bahta, 2021).

Existing international and national studies focused on crops, grass yield, the productivity of meat, milk, and wool, and the fertility of large livestock (Sheaffer et al., 1992; De Rensis and Scaramuzzi, 2003; Parry et al., 2005; Alves et al., 2013; Bodner et al., 2015; Rey et al., 2017; Salmoral et al., 2020). There are limited studies on the livestock sector, especially from the three pillars of sustainability (natural, economic, and social). Therefore, this study assessed adaptation coping strategies and resilience of agricultural drought in South Africa for the sustainability of the livestock sector.

## 2 Adaptation, resilience, and sustainability

Adaptation refers to improving resilience and reducing households' vulnerability when responding to agricultural drought impacts. Burton et al. (2002) defined adaptation as the ability of economic, environmental, and social systems to adjust to change and cope with the consequences of agricultural drought. Agriculture, including livestock, sustainability refers to the state in which agricultural production levels are maintained within the ecosystem's capacity, while also supporting and utilizing sustainability indicators that cover the three pillars of sustainable development (environmental, economic, and social) (Kajikawa, 2008). Resilience thinking, on the other hand, sees sustainability as a process of studying how to keep a system running in the face of adversity, such as agricultural drought (Folke et al., 2002; Duru and Therond, 2015).

**Figure 1:**
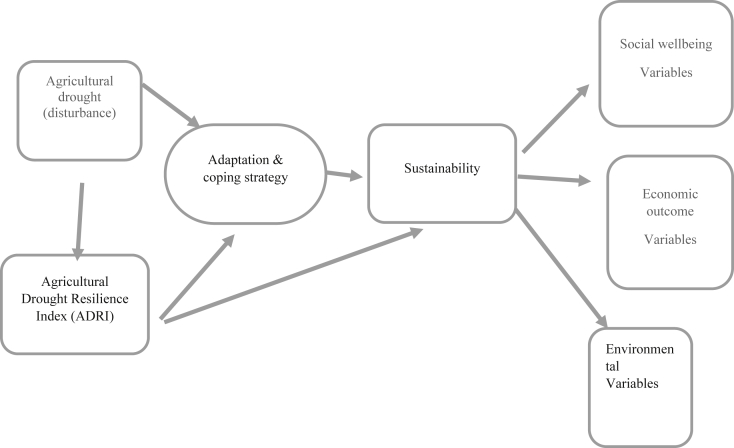
Analytical framework of the study. Source: Author's Compilation.

Following Van de Kerk and Manuel (2012); Carruthers and Hood (2004), and Diener and Biswas-Diener (2002), the three pillars of sustainability, namely social wellbeing variables (relatives, social network and part of cooperative), economic variables (access to resources (credit), other business beside farming, and types of enterprise), and environmental variables (access to land, access to water and climate change occurrence and intensity) were incorporated in this study. Besides the three pillars of sustainability, adaptive capacity variables (perception, source of income, and migration) were included. The theoretical and empirical framework applied in this study is explained in Figure 1.

## 3 Methodology

### 3.1 Study area

**Figure 2:**
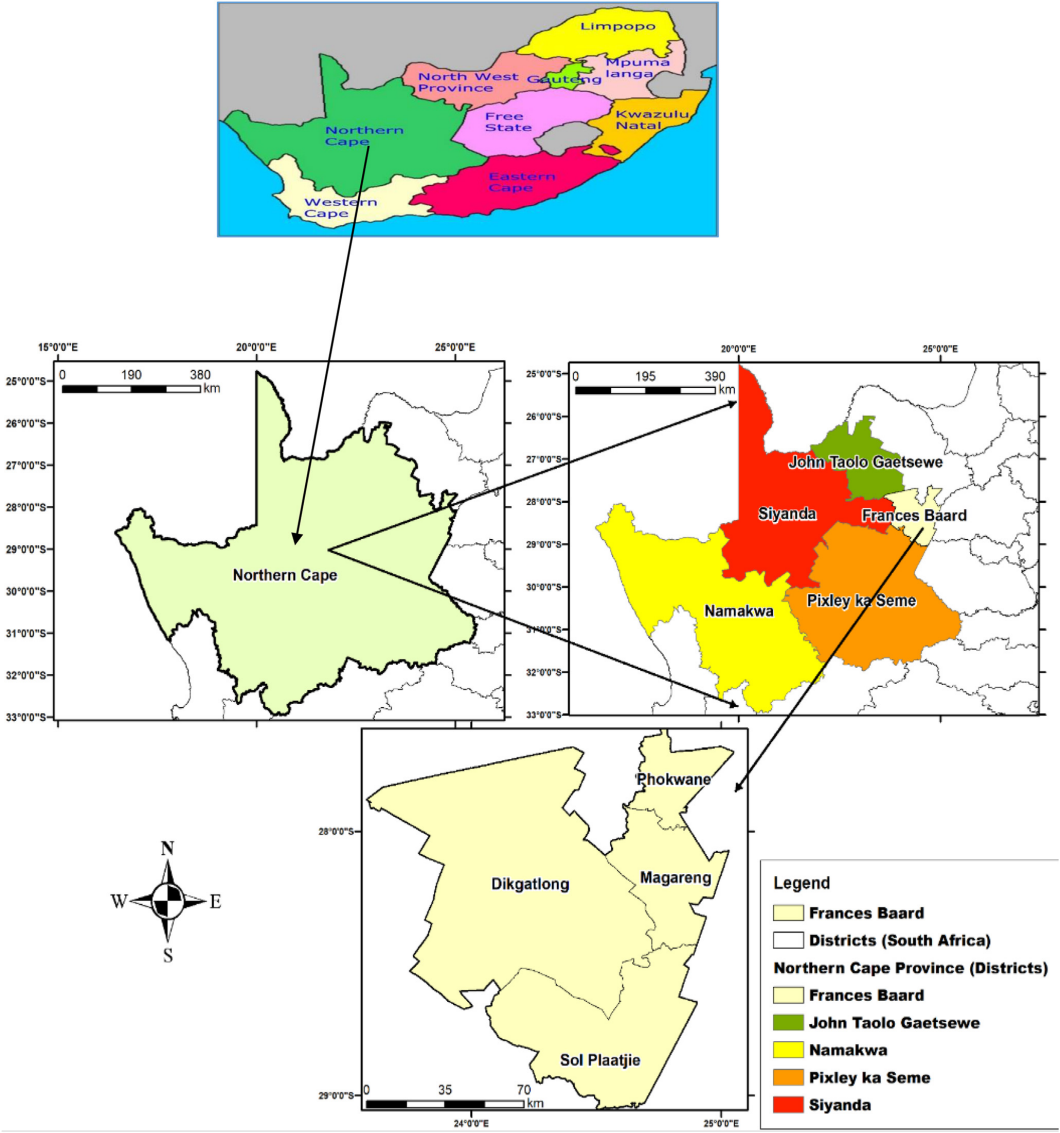
Maps of South Africa highlighting the Northern Cape Province, District municipalities of the Northern Cape, and the four local municipalities of Frances Baard District Municipality. Source: FBDM (2019).

The research was carried out in South Africa's Northern Cape Province. The Northern Cape Province is located in South Africa's northwestern corner. Frances Baard (12 800 km2), John Taolo Gaetsewe (27 300 km2), Namakwa (126 900 km2), Pixley Ka Seme (103 500 km2), and ZF Mgcawu (102 500 km2) are the province's five district municipalities. The research was carried out in the Frances Baard district municipality (FBDM) in the eastern part of the Northern Cape Province (Figure 2). Dikgatlong (2 377.6 km2), Magareng (1 541.6 km2), Phokwane (833.9 km2), and Sol Plaatje (1 877.1 km2) are the four local municipalities that make up the Frances Baard District Municipality (FBDM, 2018).

The province is divided into semi-desert and desert sections, with a hot and arid climate. Summers in the Northern Cape is hot (between 34 and 40 °C) while winters are cold (nightfall temperatures below 0 °C with frost). The climate is dry and unforgiving due to minimal rainfall (average annual precipitation of 200 mm) (Von Maltitz and Bahta, 2021).

Due to the considerable differences in climate throughout the district municipalities, the Northern Cape Province produces a diverse range of agricultural products. Livestock production continues to be the most popular business, with over 75% of agricultural households focusing only on animal production (Statistics South Africa, 2016). According to the Department of Agriculture, Forestry and Fisheries (DAFF) (2018), the Northern Cape produces 7% of the country's goats, 1.4% chickens, 24% sheep, and 4% cattle.

The agricultural sector has been decimated by the recent drought in the Northern Cape Province, and recovery has been delayed or non-existent. The livestock business has been put under tremendous strain due to a scarcity of fodder and water (DAFF, 2018). Smallholder farmers' suffering was exacerbated by a number of reasons, including insufficient grazing, a shortage of water, a scarcity of resources, and a lack of land ownership. Most smallholder farmers, according to Matlou and Bahta (2019), are vulnerable to agricultural drought. Lack social networks, lack of cooperatives, lack of access to financing, and lack of government help during droughts, contributed to their lack of drought resilience.

### 3.2 Study design

Mixed-method approach combines both qualitative and quantitative research approaches to collect relevant data employed. Through this mixed method, the following information collected socio-economics characteristics, adaptive capacity, consumption, production, social wellbeing, economic and environmental variables. According to Lieber (2009), qualitative approaches offer the advantage of giving researchers the context of the research environment and the human element, resulting in comprehensive data that quantitative methods cannot establish. Quantitative approaches, on the other hand, are concerned with gathering information and determining the relationship between variables. The combination of the two approaches gives comprehensive, in-depth study data with relevant conclusions and recommendations. As part of the standard protocol for conducting the study, meetings were held with stakeholders in the Northern Cape Province, which included smallholder livestock farmers, the African Farmers Associations of South Africa (AFASA), Northern Cape Department of Agriculture, Forestry and Fisheries, and the Department of Rural Development and Land Reform. The study's objective was explained at the meeting, and all participants agreed to participate voluntarily. A questionnaire was used and included continuous and categorical data, which comprised socio-economics characteristics, adaptive capacity, consumption, production, social wellbeing, economic and environmental variables. Face-to-face interviews with smallholder livestock producers in the Northern Cape Province of South Africa were conducted from October to December 2020, using a structured questionnaire to acquire primary data. The University of the Free State provided ethical clearance. Ethical clearance was obtained from the University of the Free State.

### 3.3 Sampling procedure and analytical technique

A multi-stage sampling technique was used to perform the survey. The Northern Cape Province was purposefully picked in the first round because it represented South Africa's primary livestock-producing province. The province was also declared a disaster region by the South African government (Maré et al., 2018; Tandwa, 2018). The FBDM was chosen at random in the second stage of the sample via balloting. Phokwane, Magareng, Sol Plaatjie, and Dikgatlong were purposefully chosen as the key livestock-producing municipalities within FBDM. Finally, the sample frame was drawn from a list of smallholder farmers identified and aided throughout the 2015/2016 crop season (Table 1). According to the Northern Cape Department of Agriculture, Forestry and Fisheries (2020), the four local municipalities assisted 878 smallholder livestock farmers registered for assistance from the local government. The government helped by providing animal feed and medication, improving access to agricultural loans and farm input, and increasing smallholder farmers' participation in agricultural drought resilience efforts by providing training and disseminating information. Based on a simple random sampling formula of Cochran (1997) and Bartlett et al. (2001), 217 smallholder livestock farmers were selected from the 878 assisted farmers.

**Table 1:** Sampling procedure and smallholder livestock farmers received assistance.

Municipalise	Number smallholder farmers	Share (number farmers/total)%	Sample (Percentage ∗total sample size [217])
Dikgatlong	351	40	87
Magareng	120	14	30
Sol Plaatje	141	16	35
Phokwane	266	30	65
Total	876	100	217

Note: The asterisk (∗) represents multiplication.

Sources: Northern Cape Department of Agriculture, Forestry, and Fisheries (NDAFF) (2020).

The Cochran's (1997) sample size formula was applied to determine the correct sample size (Equation 1):

(1) $$\text{Sample ​size}=\frac{{\left(\text{z}\right)}^{2}\text{∗}\left(\text{w}\right)\left(\text{m}\right)}{\left(\text{x}\right)²}$$

where z is the level of confidence/Alpha level, w and m are the estimates of the variance of the population, and x the margin of error (5% (0.05)). Therefore (Equation 2):

(2) $$\text{Sample}\;\text{size}=\frac{{\left(1.65\right)}^{2}\text{∗}\left(0.515\right)\left(0.515\right)}{\left(0.05\right)²}$$

Samplesize=288.83

However, Sample size when exceeding 5% of the population, Cochran's (1997) correctional formula should be applied (Eqs. (3) and (4)):

(3) $$\text{N}1=\frac{\text{Sample}\;\text{size}}{1+\left(\text{N0}/\text{population}\right)}$$

(4) $$N1=\frac{288.83}{1+\left(288.83/878\right)}$$

N1=217

### 3.4 Analytical procedures

#### 3.4.1 Agricultural drought resilience index (ADRI)

The agricultural drought resilience indices (ADRI) were calculated using the concept and scale of Walsh-Dilley et al. (2013). The ADRI was calculated in four steps: (i) selecting resilience indicators; (ii) normalizing the selected indicators; (iii) creating weights; and (iv) aggregating the final resilience index. Principal Component Analysis (PCA) was used to calculate the weights for each resilience indicator. According to the proportion variance explained by each indicator, we assigned weights based on PCA. We also used expert judgment to assess the PCA weights. The weighting was done to see any possible association between the indicators and avoid overlapping components (Cutter et al., 2008; Matlou et al., 2021). The ADRI has been applied for crops by Banda et al. (2016) and the livestock sector by Matlou and Bahta (2019), Matlou et al. (2021), and Myeki and Bahta (2021).

The ADRI was constructed using the following resilience indicators: livestock production in a normal year (WnPn), livestock production in agricultural drought (WdPd), the number of months a household consumes food produced by the household in a normal year (WcnMn), and the number of months a household consumes food produced by the household in agricultural drought (WcdMd). PCA considers the variation in the original data or variables to reduce a large number of variables to smaller variables (Holland, 2008; Beaumont, 2012).

The four indicators (W_n_P_n_, W_d_P_d_, W_cn_M_n_, W_cd_M_d_) are aggregated into an Agricultural Drought Resilience Index (ADRI) expressed as (Equation 5):

(5) ADRI = WnPn + WdPd + WcnMn + WcdMd

where: W- each component of Eq. (5) is a weighted linear combination of the variables determined from principal components' loadings with a zero mean and unit variance.

As presented in Table 2, due to variables that measure the same construct, a high correlation among variables was observed. When considering the communalities and the initial communalities, it is clear that they are all greater than 0.30, which is a good sign. Based on the analysis of eigenvalues, one factor was extracted. The total variance explained indicates that 94.402% of the component explains the total variance. The results of the Bartlett test of sphericity are demonstrated. The results show that the null hypothesis is that the inter-correlation matrix is an identity matrix. The reduction of variables is rejected since the inter-correlation matrix did not drive from a population. The KMO statistics for the model amounted to 0.636, and the Bartlett test of sphericity was significant (p-value = 0.000 chi-square = 2224.837).

**Table 2:** PCA analysis of ADRI.

Variables	Communalities		Component factors	Corrr.ADRI
	Initial Extraction		1	
WnPn	1	0.935	0.967	0.894
WdPd	1	0.958	0.979	0.995
WcnMn	1	0.280	0.963	0.890
WcdMd	1	0.955	0.977	0.984

Cumulative (%) 94.402.

KMO sampling adequacy test = 0.636.

Bartlett's sphericity test is significant at p = 0.0000; chi-square = 2224.837.

Source: Author's Estimation (2020).

**Figure 3:**
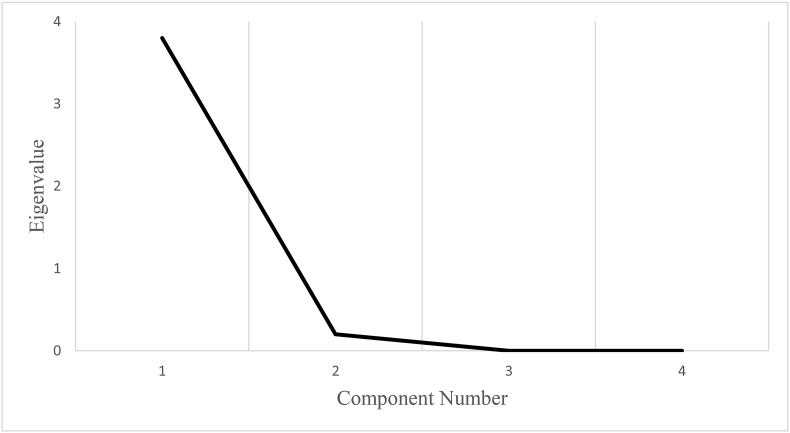
Scree plot of ADRI. Source: Author's Estimation (2020).

Eigenvalues are the variances of the principal components. The size of the eigenvalue is used to determine the number of principal components. A scree plot was used to compare the size of the eigenvalues visually (Figure 3). The first principle component has an eigenvalue greater than 1, explain 94.402% on the variation in the data. The scree plot shows that the eigenvalue starts to form a straight line after the first principle component. Therefore, 94.402% is an adequate amount of variation explained in the data. As a result, the first principle component was utilized.

ADRI can be written as:

(6) ADRI = WnPn ∗ 0.967 + WdPd ∗ 0.979 + WcnMn ∗ 0.963 + WcdMd ∗ 0.977

Based on Eq. (8), ADRI calculated incorporating the data obtained from the interview related to production and consumption.

#### 3.4.2 Structural equation modeling

A structural equation model was applied in this study (Table 3). The model applies a factor analysis-style model to measure latent variables via observed variables while also utilizing a regression-style model to characterize the connection between the latent variables (Bollen, 1989; Alinovi et al., 2010a, 2010b). Using ADRI as an outcome variable, ADRI regressed against the three pillars of sustainability (social wellbeing variables, economic outcome variables, and environmental economy variables) with adaptive capacity, empirically expressed as (Equation 7):

(7) ADRI = f (social wellbeing variables, economic variables, environment variables)

**Table 3:** Description of variables.

Variables	Descriptions	
Outcome variable		
Agricultural Drought Resilience Index (ADRI)		
Explanatory variables	Sub-variables	Descriptions
Social wellbeing variable (SWV)	Relationship (Relative)	Interpersonal relationship (neighbours, family and other relationships) (How many members of your family/relatives are you staying with?)
	Social farm actualization (social network)	Positive comfort level with society (What role does social network play in the fight against drought?)
	Social farm integration (part of cooperation)	Feeling like a part of the farming community (Are you part of a cooperative? (Yes/No)).
Economic outcome variables (EOV)	Resources	Assets (Do you own any of the following: assets, land, livestock, house? (Yes/No)
	Multiple streams of income	Generating income from additional business/property (Do you have additional property/business that generates extra income to recover during drought years? (Yes/No).
	Agripreneurship (other additional types of a farm)	Entrepreneurship in agriculture (What kind of agriculture business do you run? (only animals = 1, only crops = 2, mixed farming = 3, other = 4))
Environmental variable (EV)	Land (access)	Land availability for grazing, crop production, and livestock grazing (land ownership (customary = 1, rented = 2, purchased = 3, other = 4))
	Climate (drought occurrence and intensity	Occurrence and intensity (When did the last time drought occur? (less than 12 months = 1, less than 5 years = 2, and more than 5 years = 3), (Do you think the intensity of this drought is: (worse than the previous droughts = 1; similar to the previous droughts = 2; better than previous droughts = 3)
	Access to water	Availability of sources of water (dams, rivers) (Is there a nearby water supply (river/dam) for the household? (Yes/No)).
Adaptive capacity	Perception	Perceptions regarding risk, rainfall variation (Do you believe that the climate is changing to the extent that it will affect your livestock production? (Yes/No))
	Source of income	Off-farm activities generate additional income from employment and business (How many members of your household are employed?/Is there any other business the household is doing besides farming? Yes/No), If yes, please specify
	Migration	Scarcity of employment, natural resource depletion, and deterioration of food and rural livelihoods (Is migration an adaptive option during the drought? Yes/No- what pushes them to migrate?)
	Credit	Access to credit (Do you have access to credit when you need them? (Yes/No)

Source: Author's observation (2020).

Eq. (7) is disaggregated in Eq. (8), and the detailed description of the variables is also illustrated in Table 3.

(8) ADRIi =f(SWV ( relative, social network, part of cooperative); ADC (Perception; Source of income; migration; credit), EOV (resource, multiple sources of income, agripreneurship); EV (access to land, drought Occurrence, and intensity, access to water))

## 4 Results and discussion

### 4.1 Adaptation and coping strategies

**Figure 4:**
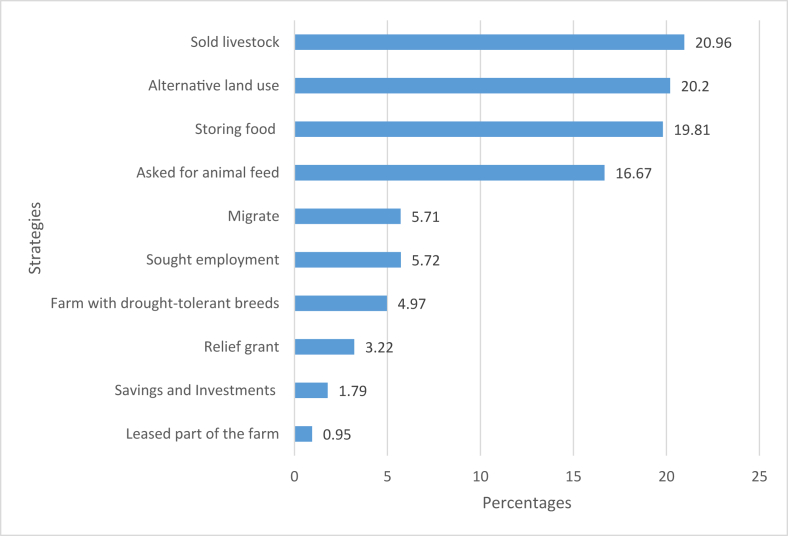
Households' adaptation and coping strategies. Source: Author's compilation based on Survey Data (2020).

Farmers must have an adaptation and coping strategy when they deal with drought. Figure 4 shows that selling livestock is the most common adaptation and coping strategy; 20.96% of the farming households sold their livestock as an adaptation and coping strategy. These findings concurred with Bahta (2020). Further, Clements et al. (2011) highlighted that farmers could get money whenever they wanted by selling their animals during droughts.

In contrast, 20.2% of the farming households utilized alternative land use (such as horticulture) as an adaptation and coping strategy, 19.81% stored food, 16.67% asked for animal feed (assistance from government/department of agriculture supplied fodder or vouchers to buy fodder), 5.72% sought employment, 5.71% migrated, 4.97% farmed with drought-tolerant breeds, 3.22% obtained relief grants, 1.79% used their savings and investments, and 0.95% leased their farms (Figure 4).

### 4.2 The three pillars of sustainability with adaptive capacity

The resilience of the livestock sector to agricultural drought in the Northern Cape Province of South Africa was assessed using adaptive capacity (ADC) as well as the three pillars of sustainability, namely social wellbeing (SWV), economic wellbeing (EOV), and environmental wellbeing (EV); Table 4 shows the regression results. The results indicated that all three pillars of sustainability with adaptive capacity have a positive and significant impact on households' ADRI. Social wellbeing (ß = 0.976), economic outcome (ß = 0.144), environmental (ß = 0.155), and adaptive capacity (ß = 0.008) variables contributed to the regression model. The Variance Inflation Factor (VIF) statistics for all the models indicated no multicollinearity problem in the analysis (Table 4). This meant that the greater the participation of smallholder farmers in social networks and cooperatives, the greater their resilience to agricultural drought.

**Table 4:** Regression analysis smallholder livestock farmers’ agricultural drought resilience index (ADRI) using adaptive capacity (ADC), social wellbeing (SWV), economic wellbeing (EOV) and environmental wellbeing (EV).

ADRI	Unstandardized Coefficients	Standardized Coefficients		Sig.	
	B	ß	Std. error		VIF
Constant	0.744		0.194	0.000∗∗∗	
SWV	1.015	0.976	0.009	0.000∗∗∗	1.55
EOV	0.848	0.144	0.050	0.000∗∗∗	1.67
EV	1.205	0.155	0.068	0.000∗∗∗	1.86
ADC	0.025	0.008	0.028	0.000∗∗∗	1.90
R^2^ 0.988					

∗∗∗ = significant at 1% level.

Source: Authors' estimation (2020).

Furthermore, the stronger a farming household's resilience to agricultural drought and adaptive capacity was the more resources, income, access to land, water, credit, and additional types of the farm they possessed. These findings are consistent with Matlou and Bahta (2019), who found that belonging to a cooperative positively impacted farmers' resilience to agricultural drought. Keil et al. (2008) found involvement in a number of village organizations positively impacted farmers' resilience to agricultural drought. Furthermore, the literature suggests that resilience is essential for improving adaptive capacity (Folke et al., 2002).

#### 4.2.1 Social wellbeing

Social networks can be either informal (e.g., church groups) or formal (e.g., farmers' associations) (Wongbusarakum and Loper, 2011). Iglesias et al. (2007) and Hassen (2008) highlighted that when farmers participate in local institutions, their resilience to agricultural drought is enhanced because members of the social network could help each other and access additional resources. The lack of effective social networks contributes to social vulnerability during agricultural drought. As shown in Table 5, assistance from relatives or being part of cooperatives positively impacted households' ADRI. This finding is consistent with Matlou and Bahta (2019), Andersen and Cardona (2013), Banda et al. (2016), and Keil et al. (2008). They found that households receiving support from relatives or cooperatives tend to be more resilient than those not receiving support or were not members of cooperatives. However, social networks had a negative and significant effect irrespective of the negative signs. Social networks contributed more (ß = -0.202) to the regression model than cooperatives (ß = 0.123) or relatives (ß = 0.007) (Table 5).

**Table 5:** Estimation of smallholder livestock farmers’ agricultural drought resilience with regard to adaptive capacity (ADC) social wellbeing (SWV), economic wellbeing (EOV) and environmental wellbeing (EV).

Indicators	Unstandardized Coefficients		Standardized Coefficients	Sig.
	B	Std. error	ß	
Constant	0.744	0.194		
*SWV*				
Relative	0.002	0.021	0.007	0.920
Social network	-2.020	2.642	-0.202	0.445
Cooperatives	1.262	2.716	0.123	0.005∗
*EOV*				
Resources/Wealth	0.504	0.197	0.193	0.011∗∗
Multiple stream income	0.132	0.187	0.053	0.479
Agripreneurship	0.096	0.076	0.093	0.204
*EV*				
Land	1.291	0.000	0.152	0.064∗
Climate	0.022	0.030	0.059	0.465
Water	-0.002	0.097	-0.001	0.001∗∗∗
*ADC*				
Perception	-0.154	0.057	-0.181	0.007∗∗∗
Income source	-0.235	0.132	-0.122	0.077
Credit	-0.541	0.155	0.250	0.001∗∗∗
Migration	0.059	0.113	0.037	0.603

∗∗∗ = significant at 1% level; ∗∗ = significant at 5% level; ∗ = significant at 10%.

Source: Authors' estimation (2020).

#### 4.2.2 Economic outcome

Regarding economic outcome, resources, multiple streams of income, and agripreneurship indicators positively impacted households' ADRI. As shown in Table 5, resource (ß = 0.193), multiple streams of income (ß = 0.053), and agripreneurship (ß = 0.093) contributed to the regression model. The marginal effect values for resource, multiple income, and agripreneurship indicated that up to a certain point, for one increase in economic outcome variables, the probability of the household becoming resilient to agricultural drought increased by 0.193, 0.053, and 0.093, respectively, while holding all other factors constant. Ciani and Romano (2004) and Melketo et al. (2021) found similar results. The authors discovered that economic variables are positively and significantly associated with the resilience index. If a household has numerous appropriate resources, the impact of a shock is decreased because not all resources are affected simultaneously. According to Iglesias et al. (2007), agricultural drought may lower forage production in animal production, resulting in changes in ideal farming systems and a loss of rural income.

#### 4.2.3 Environmental variables

Saisana and Philippas (2012), Cruz and Manata (2020), and Community Tool Box Team (2021) highlighted that environmental factors include water and land. Thus, access to land and water was included under environmental variables. Access to land and duration and intensity of drought positively impacted households' ADRI. Fellmann (2012) highlighted that agricultural drought impacted the agricultural sector, including the livestock sector, in multiple ways, such as frequency and intensity of extreme weather events. As shown in Table 5, land (ß = 0.152), climate change (ß = 0.059), and access to water (ß = -0.001) contributed to the regression model. Access to water nearby had a negative and significant effect on resilience. In the face of water shortage, this meant that agricultural diversification could have aided respondents in coping better during the drought by providing additional income to support their core farming enterprises. This was in line with Bahta's findings (2020).

Agricultural drought, according to Gitz and Meybeck (2012), reduces water availability and makes some pastures inaccessible, increasing the demand for feed. As a result, feed prices rise, forcing livestock owners to sell their animals. Cattle prices fall due to increased livestock sales during a period of low demand, requiring farmers to sell even more to buy feed. These price effects have a negative impact on farm and household income and assets. Furthermore, they depreciate assets.

#### 4.2.4 Adaptive capacity

Increasing adaptation ability and reducing household vulnerabilities to agricultural drought are two ways to improve resilience (either environmental, economic, or social). The results in Table 5 show that migration and access to credit had a positive impact on households' resilience. Migration (ß = 0.037), credit (ß = 0.250), perception (ß = -0.181), and source of income contributed (ß = -0.122) to the regression model. The results of the marginal effect on the source of income revealed that, up to a certain point, a decrease in the source of income reduces the likelihood of farming households' resilience to unfavourable effects of agricultural drought by 0.122, while all other factors affecting resilience remain constant.

According to the results of the marginal effect on the variable credit, an increase in credit access raises the likelihood of a farming household becoming resilient to agricultural drought by 0.250. This could be due to the fact that finance allows smallholder farmers to diversify their livelihood strategies beyond livestock production and provide better access to off-farm and non-farm income-generating activities, improving their resilience. This finding is in line with Matlou and Bahta (2019), Andersen and Cardona (2013), and Banda (2016), who found that credit-enabled households were more resilient.

## 5 Conclusion and recommendations

The study aimed to assess the adaptation and coping strategies, the resilience of agricultural drought, and its implication for the sustainability of livestock farming in the Northern Cape Province of South Africa. The study was crucial to elucidating production and sustainability on a long-term basis and developing future interventions. The results found that 21% of the livestock farming households sold their livestock, 20% used alternative land use, 20% stored food, 17% asked for animal feed, 6% sought employment, 6% migrated, 5% farmed with drought-tolerant breeds, 3% received relief grants, 2% used their savings and investments, and 1% leased their farms as adaptation and the coping strategies. When natural, economic and social sustainability was viewed as a resilience process, the three pillars positively and significantly impacted households' agricultural drought resilience. This implied that the more smallholder farmers participated in social networks and cooperatives, the higher the resilience to agricultural drought. Further, the more resources, income, access to land, access to water, access to credit and additional types of farming, the higher the households’ resilience to agricultural drought and adaptive capacity. Thus, the three pillars of sustainability are crucial for enhancing the resilience and adaptability of smallholder livestock farmers. The study recommends that government aid reduce vulnerability to agricultural drought via access to agricultural credit and encourage farmers to be part of social networks and cooperatives. Additionally, the government could improve access to land and water rights to boost the resilience of smallholder farmers to agricultural drought. This could be achieved through collaboration and coordination among all role players.

The study used primary data to assess the adaptation and coping strategies and agricultural drought resilience for only smallholder livestock farming households. There were some delays in collecting data caused by the Covid-19 pandemic, and a language barrier was also a limitation. Afrikaans and Setswana (local South African languages) are the most widely spoken languages in the Northern Cape, making communication challenging between the researcher and the respondents.

Future research could, for example, look into adaptation and coping strategies, the resilience of agricultural drought on mixed farming, which is outside the reach of this study. Such research could contribute to identifying specific adaptation and coping strategies for both crop and livestock farmers.

## Declarations

### Author contribution statement

Yonas T. Bahta: Analyzed and interpreted the data.

Vuyiseka A. Myeki: Contributed reagents, materials, analysis tools or data.

### Funding statement

This work was supported by the project “Household resilience to agricultural drought in the Northern Cape province of South Africa” Contract Number/Project Number (TTK170510230380) of the 10.13039/501100001321National Research Foundation (NRF), Thuthuka.

### Data availability statement

Data will be made available on request.

### Declaration of interests statement

The authors declare no conflict of interest.

### Additional information

No additional information is available for this paper.
